# Phosphorylation of Plant Microtubule-Associated Proteins During Cell Division

**DOI:** 10.3389/fpls.2019.00238

**Published:** 2019-03-11

**Authors:** Tereza Vavrdová, Jozef ˇSamaj, George Komis

**Affiliations:** Department of Cell Biology, Centre of the Region Haná for Biotechnological and Agricultural Research, Faculty of Science, Palacký University Olomouc, Olomouc, Czechia

**Keywords:** microtubules, microtubule-associated proteins, mitotic spindle, phragmoplast, protein kinase, protein phosphatase

## Abstract

Progression of mitosis and cytokinesis depends on the reorganization of cytoskeleton, with microtubules driving the segregation of chromosomes and their partitioning to two daughter cells. In dividing plant cells, microtubules undergo global reorganization throughout mitosis and cytokinesis, and with the aid of various microtubule-associated proteins (MAPs), they form unique systems such as the preprophase band (PPB), the acentrosomal mitotic spindle, and the phragmoplast. Such proteins include nucleators of *de novo* microtubule formation, plus end binding proteins involved in the regulation of microtubule dynamics, crosslinking proteins underlying microtubule bundle formation and members of the kinesin superfamily with microtubule-dependent motor activities. The coordinated function of such proteins not only drives the continuous remodeling of microtubules during mitosis and cytokinesis but also assists the positioning of the PPB, the mitotic spindle, and the phragmoplast, affecting tissue patterning by controlling cell division plane (CDP) orientation. The affinity and the function of such proteins is variably regulated by reversible phosphorylation of serine and threonine residues within the microtubule binding domain through a number of protein kinases and phosphatases which are differentially involved throughout cell division. The purpose of the present review is to provide an overview of the function of protein kinases and protein phosphatases involved in cell division regulation and to identify cytoskeletal substrates relevant to the progression of mitosis and cytokinesis and the regulation of CDP orientation.

## Introduction

Owing to their sedentary life style and their encasement within the barriers of rigid cell walls, plant cells adopted unique mechanisms for regulating fundamental eukaryotic processes such as cell division and cell division plane (CDP) orientation establishment (reviewed in Buschmann and Zachgo, 2016). In this respect, plant cells developed unique microtubule-based cytoskeletal structures which underlie the above processes. CDP orientation is marked by a plant-specific cortical microtubule ring, the preprophase band (PPB; Pickett-Heaps and Northcote, 1966) which determines spindle positioning (Schaefer et al., 2017) and coincides with the plane of cell plate deposition during cytokinesis (cell plate fusion site; CPFS; Marcus et al., 2005). The PPB exhibits a progressive narrowing and finally it disassembles shortly after the mitotic spindle is formed; however, the cortical site which was occupied by the PPB is marked by specific proteins throughout mitosis in a continuous or intermittent manner (e.g., Buschmann et al., 2006; Walker et al., 2007).

The plant mitotic spindle starts to assemble before nuclear envelope breakdown and in contrast to the mammalian or yeast spindle, and it forms in the absence of microtubule organizing center (reviewed in Buschmann and Zachgo, 2016).

Cytokinesis is hallmarked by the formation of another plant specific microtubule machinery, the phragmoplast. It is formed at the end of telophase between the reconstituting daughter nuclei. It comprises two sets of antiparallel microtubules, and it expands centrifugally toward the cell periphery. During its expansion, it guides the deposition of the cell plate until the latter merges with the parent cell wall, after which the phragmoplast disintegrates (Chen et al., 2018).

**Figure 1:**
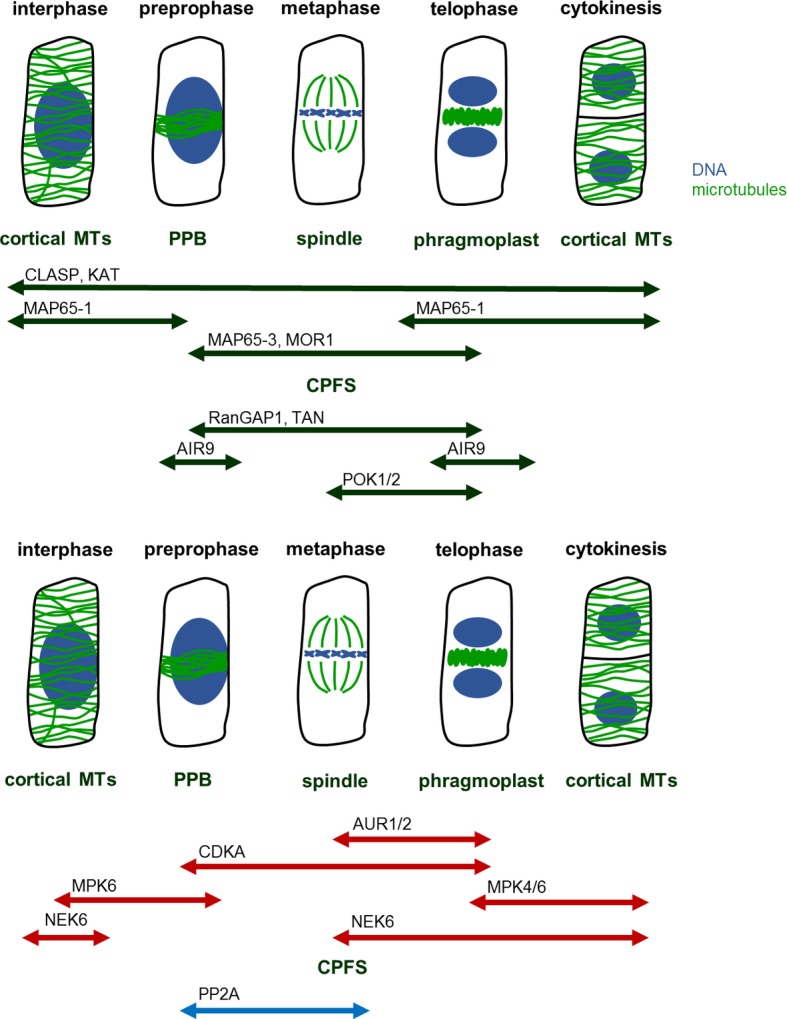
MAPs, kinases, and phosphatases regulating mitotic MT structures. Mitosis begins with preprophase, in which cortical MTs reorganize in preprophase band (PPB). PPB disassembles at the onset of metaphase, during which spindle forms. At this time, the former site of PPB remains marked as a future cell plate fusion site (CPFS) by various MAPs. After segregation of chromatids, at the late anaphase, phragmoplast begins to form at the center of cell. Phragmoplast serves as a scaffold for building cell plate and as the construction continues, phragmoplast expands until it reaches CPFS. At the end, in two daughter cells, MTs rearrange into cortical microarrays. Abbreviations: AIR9, auxin-induced root cultures; AUR, aurora kinase; CDKA, cell division kinase A; CLASP, cytoplasmic linker associated protein; CPFS, cell plate fusion site; KAT, katanin; MAP65, microtubule-associated protein 65; MOR1, microtubule organization 1; MPK, mitogen-activated protein kinase; MT, microtubule; NEK, never in a mitosis A-related kinase; PP2A, protein phosphatase type 2A; POK, phragmoplast orienting kinesin; PPB, preprophase band; RanGAP1, Ran GTPase activating protein; TAN, tangled.

Throughout the cell cycle, precise temporal and spatial regulation of microtubule organization and dynamics is required for the formation, proper function, and structural transitions of these cytoskeletal structures (Dhonukshe and Gadella, 2003). Such regulation is achieved *via* microtubule-associated proteins (MAPs) involved in microtubule organization and dynamics. Among these proteins belong motor proteins from the kinesin (Müller et al., 2006; Lipka et al., 2014; Buschmann et al., 2015; de Keijzer et al., 2017) and the myosin superfamilies (Wu and Bezanilla, 2014), plus end-binding proteins and microtubule crosslinkers (Mao et al., 2005; Beck et al., 2010; Kohoutová et al., 2015; Lin et al., 2019). Many of such proteins exhibit a cell cycle dependent localization to mitotic and cytokinetic microtubule systems (Figure 1), and at large this is differentially regulated by protein kinases and phosphatases which become activated/deactivated in a similar cell cycle dependent manner.

Many kinases were directly shown to associate with cytoskeletal systems (Weingartner et al., 2001; Samaj et al., 2002, 2004; Lee et al., 2003; Oh et al., 2005, 2012) and indirect pharmacological (e.g., Binarova et al., 1996; Ayaydin et al., 2000) and subsequently more targeted studies (e.g., Mao et al., 2005; Brumbarova and Ivanov, 2016), establishing the functional reciprocity between protein kinases and cytoskeletal components. Plant microtubule systems can be targeted for phosphorylation-pendant regulation of their components after environmental stimulation (e.g., Ban et al., 2013; Bhaskara et al., 2017), or in a developmental context, which is the aim of this review.

## Involvement of Maps in the Organization of Mitotic Structures

From numerous plant proteins related to the regulation of microtubule organization and dynamics, some have been inadvertently associated with the progress of mitotic and cytokinetic microtubule arrays and were shown to be regulated by reversible phosphorylation. These proteins are involved in all aspects of microtubule organization and dynamics.

**Figure 2:**
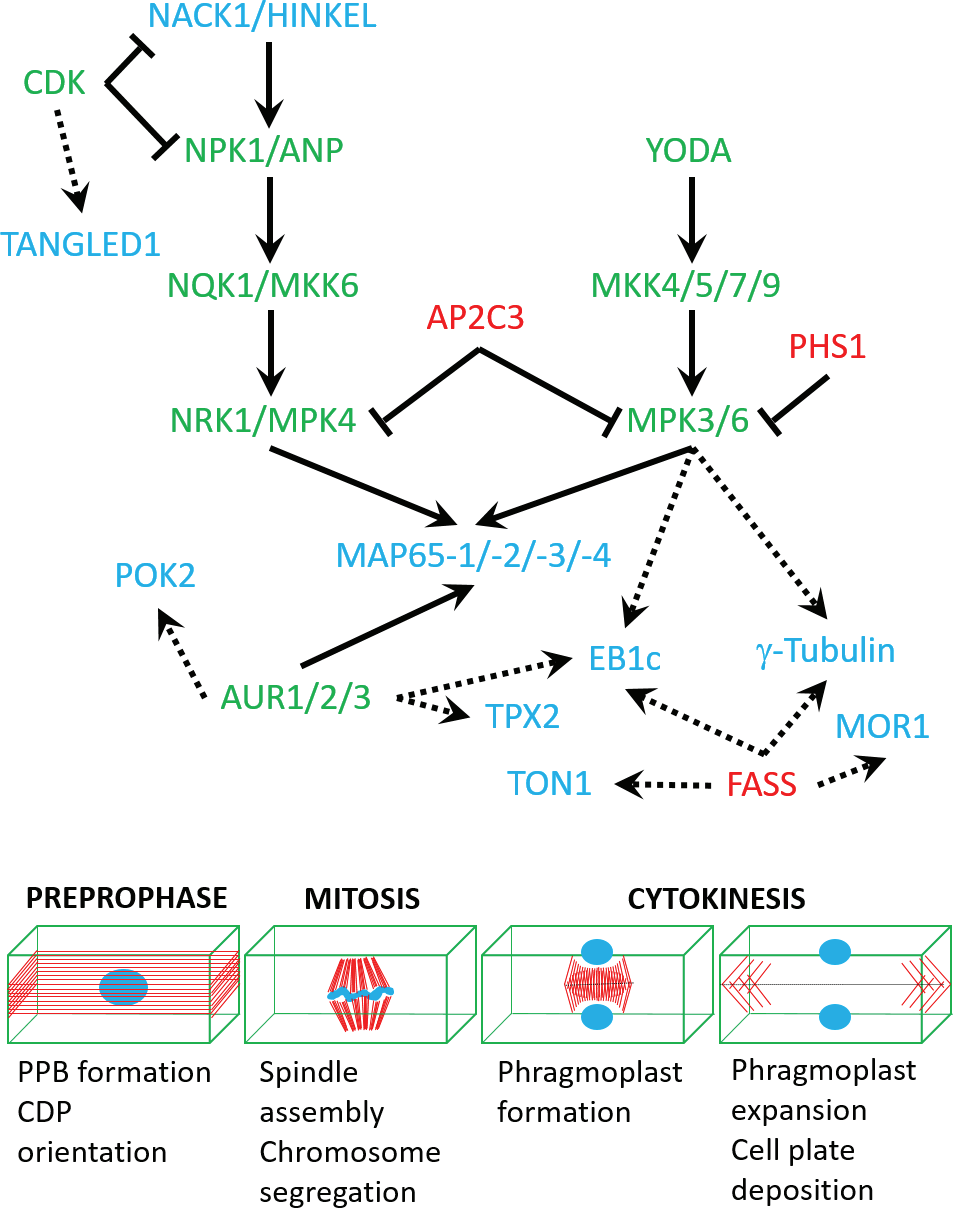
A speculative network of protein kinases (green), phosphatases (red), and targeted cytoskeletal proteins (blue) based on either published interaction studies (full arrows) or in silico predictions (dashed arrows; see text for more details). Lower panel shows mitotic stages which are regulated by the above network of interactions.

Microtubule nucleation factors such as γ-tubulin and TPX2 (targeting protein for Xklp2) are essential for spindle formation and the establishment of spindle bipolarity (Petrovská et al., 2013), and it was suggested that they are regulated by mitogen-activated protein kinase (MAPK, MPK) and/or Aurora kinase (AUR) phosphorylation (Petrovská et al., 2012; https://string-db.org/cgi/network.pl?taskId=f13kHLYXYV1W). It is likely that γ-tubulin interacts with the FASS B″ subunit of protein phosphatase 2A (https://string-db.org/network/3702.AT5G18580.1; Figure 2). Notably, *fass* mutants exhibit altered geometry of microtubule nucleation at least in interphase microtubule arrays (Kirik et al., 2012).

Microtubule dynamics are largely controlled by plus end binding proteins, including end-binding protein 1 isoforms (EB1a, b, and c; Komaki et al., 2010), SPIRAL1 (Sedbrook et al., 2004), CLASP (Ambrose et al., 2007), and GPT1 and 2 (growing plus-end tracking 1 and 2; Wong and Hashimoto, 2017). The plant-specific isoform EB1c was shown to be phosphorylated by MPK6 (Figure 2; Kohoutová et al., 2015), however, without apparent functional implications. Similarly, phosphorylation of CLASP was demonstrated only in the context of conditional signaling (Brumbarova and Ivanov, 2016).

Microtubule bundling *via* the MAP65 proteins is essential for the formation of the central spindle, its subsequent reorganization into phragmoplast and for support of its centrifugal expansion (Chang et al., 2005; Smertenko et al., 2008, 2018; Herrmann et al., 2018). From the nine members of the *Arabidopsis* MAP65 family, only MAP65-1, -2, -3, and -4 have been associated with the progression of mitosis and cytokinesis (Chang-Jie and Sonobe, 1993; Chan et al., 1999; Caillaud et al., 2008). MAP65-1 and MAP65-2 are nonessential as proven by the absence of cytokinetic phenotypes of single or double mutants (Lucas and Shaw, 2012). On the other hand, MAP65-3 and MAP65-4 appear to be essential for cytokinesis, in an additive manner (Müller et al., 2004; Li et al., 2017). MAP65-1 and MAP65-2 proteins differentially colocalize with microtubules and mediate their bundling during interphase and preprophase (Murata et al., 2013; Zhou et al., 2017). However, they are excluded from the mitotic spindle until telophase. This suggests that their colocalization with microtubule structures is subjected to temporal control during cell division (Gaillard et al., 2008). One possible mechanism controlling the differential localization of MAP65 proteins with mitotic microtubule systems is proteolytic degradation, since several *map65* genes harbor a “destruction box” motif, which is a target for the ubiquitin degradation pathway. More importantly, at least MAP65-1, -2, and -3 are targeted for phosphorylation in their C-terminal microtubule binding domains by several kinases with cell cycle functions, such as cyclin-dependent kinases [CDKs (Smertenko et al., 2006), MAPKs (Kosetsu et al., 2010; Smékalová et al., 2014), and AURs (Boruc et al., 2017)]. Generally, phosphorylation downregulates microtubule binding of MAP65s; therefore, it may represent the means to abolish their localization from the mitotic spindle. This is supported by mutagenesis studies, showing that change of the consensus CDK-targeted site of MAP65-1 causes its localization at the mitotic spindle (Mao et al., 2005). As mentioned earlier, MAP65-1 and presumably MAP65-2 are nonessential for the mitotic and cytokinetic progress (Lucas and Shaw, 2012), and they may affect spindle and phragmoplast formation only when artificially overexpressed (Mao et al., 2005). MAP65-3, on the other hand, is essential for cell plate formation, since its genetic depletion results in the formation of giant, multinucleated cells with incomplete cell walls. Similar cytokinetic phenotypes have been observed in *anp2anp3* and *mpk4* mutants, which are related to MAPK signaling. The above mutants show reduced but not abolished MAP65-3 expression (Beck et al., 2010). In this case, it is speculated that the cytokinetic phenotype of *anp2anp3* and *mpk4* mutants maybe partially attributed to reduced phosphorylation of MAP65-3 (Beck et al., 2010). MAP65-4 alone has negligible cytokinetic phenotypes when depleted but contributes to the *map65-3/pleiade* phenotype in double mutants (Li et al., 2017). Its spatial localization coincides with that of MAP65-3 at the PPB and the phragmoplast midzone. However, MAP65-4 exhibits persistent localization at the cortical division zone throughout mitosis, unlike MAP65-3 (Li et al., 2017). Although the cytokinetic role of MAP65-4 was just recently described, it is also likely to be regulated by phosphorylation. Its carboxyl-terminal region harbors proline-directed serine or threonine residues, which are predicted targets of CDKs and MAPKs (based on prediction using GPS2.1.2; Xue et al., 2008). It is also predicted to interact with AUR (https://string-db.org/cgi/network.pl?taskId=qRfSrQ0dHJdK; Figure 2).

Several Arabidopsis microtubule motors of the kinesin superfamily, namely those related to the progress of mitosis and cytokinesis were shown to be regulated by phosphorylation. One example is the kinesin-like calmodulin-binding protein (KCBP), which is involved in the tethering of phragmoplast margins to the CPFS (Buschmann et al., 2015; Buschmann and Zachgo, 2016). KCBP was shown to be regulated by phosphorylation (Day et al., 2000; Humphrey et al., 2015). The mitotic kinesin NACK1 (NPK1-activating kinesin-like protein 1) is an activator of the NPK1-NQK1-NRK1 MAPK pathway in tobacco (similarly, its *Arabidopsis* homologue HINKEL also activates the ANP-MKK6-MPK4 MAPK pathway; Nishihama et al., 2002), which is involved in the regulation of cytokinesis. The interaction between NACK1 and NPK1 is negatively regulated by CDK phosphorylation in residues of both the motor domain and the stalk region of NACK1. CDKs also target the carboxyl-terminal regulatory domain of NPK1, which is engaged in NACK1-NPK1 interactions (Sasabe et al., 2011a). Interruption of NACK1-NPK1 interaction by CDK-mediated phosphorylation abolishes the recruitment of the NPK1-NQK1-NRK1 module to the mitotic spindle. When CDK activity declines during late anaphase, then the NPK1-NQK1-NRK1 module becomes localized to the phragmoplast (Sasabe et al., 2011a).

Interactions of kinesin motors with protein kinases may be reciprocal and may convey targeted transport or activity regulation to the kinase counterpart. For example, never in a mitosis (NIMA)-related kinase 6 (NEK6) is negatively regulated by the armadillo-repeat kinesin 1 (Eng et al., 2017). Also, NACK1 (and its Arabidopsis homologue HINKEL) directly activates the NPK1 MAPKKK (and its Arabidopsis homologues ANP1, 2, and 3; Nishihama et al., 2002; Takahashi et al., 2010).

Microtubule severing by the Arabidopsis KATANIN1 (Komis et al., 2017; Panteris et al., 2018) has not been shown to be regulated by phosphorylation yet. Moreover, the p60 catalytic subunit of the katanin holoenzyme is suspected to interact with FASS (https://string-db.org/cgi/network.pl?taskId=jBYrCeTF9nPv; Figure 2). In animals, phosphorylation is a major mechanism for the exclusion of katanin activity from the mitotic spindle and connected to spindle sizing (Loughlin et al., 2011; Whitehead et al., 2013).

Apart from several MAPs that have been proven or predicted to be regulated by phosphorylation, tubulin has also been identified as a target of the atypical kinase domain of the protein phosphatase PROPYZAMIDE HYPERSENSITIVE 1 (PHS1; Fujita et al., 2013). So far, tubulin phosphorylation is related to conditional microtubule depolymerization (Ban et al., 2013), but it will be of interest to identify tubulin phosphorylation as a regulator of mitotic microtubule transitions.

## Kinases Regulating Map Activity

Since MAPs play a role in microtubule dynamics while being regulated by reversible phosphorylation, kinases and phosphatases are master regulators of microtubule reorganization throughout cell cycle. Several kinases were implicated in phosphorylating MAPs, namely CDKs, AURs, MAPKs, and NEKs.

Since CDKs are master regulators of cell cycle progression, they are also implicated cell cycle-dependent cytoskeletal reorganizations (Costa, 2017). The mode of action on CDKs on the microtubule cytoskeleton of dividing cells is not well understood, since the only known cytoskeletal CDK substrate is NACK1 kinesin (Sasabe et al., 2011a), while MAP65-1 was only shown *in vitro* to be phosphorylated by CDK (Smertenko et al., 2006). Localization observations and pharmacological and genetic evidence favor the implication of CDK in regulating microtubules. For example, *Arabidopsis* CDK was shown to colocalize with PPB, spindle, and phragmoplast (Stals et al., 1997). Second, plant CDKs were found to participate in regulating mitotic microtubule structures (Weingartner et al., 2001). Third, CDKs are known to regulate microtubule dynamics by phosphorylating animal homologues of plant MAPs (Ookata et al., 1997; Vasquez et al., 1999). Last, several plant MAPs contain a CDK phosphorylation site (Hussey et al., 2002; Smertenko et al., 2006). CDKs may be also involved in CDP orientation through the phosphorylation of cytoskeletal markers of the cell division zone such as the microtubule binding protein TANGLED1 (https://string-db.org/cgi/network.pl?taskId=4lbkQFdWZfbv; Figure 2).

Apart from CDKs, Aurora kinases are another component of cell cycle progression machinery. These Ser/Thr kinases are on lower hierarchical position than CDKs (Schecher et al., 2017). They themselves are regulated by phosphorylation and ubiquitin-dependent proteolysis (Castro et al., 2002a,b). Therefore, they are known to play a more direct role in cytoskeleton rearrangements than CDKs (Ritchey and Chakrabarti, 2014). In plants, not only do they associate with mitotic structures (Demidov et al., 2005) but also they interact with MAPs (Boruc et al., 2017). Since AUR does not possess microtubule-binding domains, its colocalization with mitotic structures is most likely related to its functional interactions with MAPs (Petrovská et al., 2012; Tomaštíková et al., 2015). Out of three members of Aurora kinase family in *Arabidopsis* (Kawabe et al., 2005), two of them, AUR1 and AUR2, are essential for regulating the orientation of formative cell divisions throughout plant ontogenesis (Van Damme et al., 2011). AUR phosphorylates MAP65-1 during metaphase (Boruc et al., 2017); however, the strength of AUR control over MAP65-1 is significantly weaker compared with the effect of another kinase, MAPK. Therefore, a hypothesis was presented, according to which the direct control of AUR over MAP65-1 is mild, yet the phosphorylation of MAP65-1 by AUR promotes phosphorylation by other kinases. This is in line with the observation that regulation of many eukaryotic proteins depends on multisite phosphorylation (Cohen, 2000; Repetto et al., 2018). Prediction studies show that other cytoskeletal regulators of mitosis and especially of CDP orientation like POK2 may interact and become regulated by Aurora kinases (https://string-db.org/cgi/network.pl?taskId=f13kHLYXYV1W; Figure 2).

MAPKs are well known to phosphorylate MAPs (Hoshi et al., 1992; Shiina et al., 1992). In *Arabidopsis*, MPK4 and MPK6 phosphorylate proteins from MAP65 family (Smertenko et al., 2006; Sasabe et al., 2011b; Smékalová et al., 2014; Zhou et al., 2017), and MPK6 also phosphorylates EB1c (Kohoutová et al., 2015). MAPKs are governed by MAPK kinases (MAPKKs), which, in turn, are regulated by MAPKK kinases (MAPKKKs). In plants, two MAPK signaling cascades were implicated in regulating microtubule dynamics during cell division and described in detail as follows. A third pathway which involves the MAPK MPK18 and the MAPK phosphatase PROPYZAMIDE HYPERSENSITIVE 1 (PHS1) is somehow elusive without knowledge on microtubule-associated substrates which may justify their role in microtubule regulation (Naoi and Hashimoto, 2004; Walia et al., 2009; Fujita et al., 2013). However, the role of PHS1 may be broader since it is presumably interacting and deactivating other MAPKs as well, including MPK3 and MPK6 (https://string-db.org/cgi/network.pl?taskId=j8pmY8S1UlbB; Figure 2).

The first MAPK cascade described to play a role in microtubule reorganization was the NACK-PQR pathway (Calderini et al., 1998, 2001; Bögre et al., 1999; Nishihama, 2001). In *Arabidopsis*, this pathway consists of ANP2/ANP3 (*Arabidopsis* nucleus and phragmoplast-localized kinase, MAP3K), MKK6, and MPK4/MPK6 (Krysan et al., 2002; Strompen et al., 2002), and it plays a crucial role during phragmoplast and cell plate formation (Takahashi et al., 2010). It affects the organization of mitotic structures *via* reversible phosphorylation of MAP65 proteins (Beck et al., 2010). Interestingly, activation of this MAP3K is negatively regulated by CDKs (Sasabe et al., 2011a). Moreover, CDKs also interfere with MAPK phosphorylating MAP65-1, since the single MAPK targeting motif in MAP65-1 overlaps with a CDK targeting site (Smertenko et al., 2006). In conclusion, this MAPK cascade controls microtubule organization and dynamics during phragmoplast and cell plate formation, and the temporal regulation of this module is facilitated by CDKs.

The other plant MAPK pathway, which is integral to cell division directionality, consists of YODA (YDA, MAP3K), MKK4/MKK5, and MPK3/MPK6. YODA is implicated in several types of asymmetrical divisions, e.g., first division of zygote and stomatal development (Lukowitz et al., 2004; Bergmann et al., 2004). However, the characterization of *YODA* mutants revealed its function in CDP orientation of regular cell divisions underlying tissue patterning of vegetative organs such as the root (Müller et al., 2010; López-Bucio et al., 2014). These observations are further supported by microscopic studies, which proved MPK6 colocalization with mitotic microtubule structures (Müller et al., 2010; Smékalová et al., 2014; Komis et al., 2018). Interaction studies showed that MAP65-1 is interacting with MPK6 and possibly phosphorylated by it (Smékalová et al., 2014). Interestingly, knockout mutants of YDA have deregulated transcript levels of CPFS markers (specifically TAN and phragmoplast orienting kinesin 1). Therefore, YODA may be involved at multiple levels of CPFS orientation (Smékalová et al., 2014).

The last family of kinases involved in regulation of mitotic microtubule structures is NEKs. This family of Ser/Thr protein kinases is highly conserved in eukaryotes, where it supervises crucial points in mitosis and cell division (O’Connell et al., 2003; Brieño-Enríquez et al., 2017). In plants, NEKs were shown to regulate cortical microtubules and, in turn, to affect cell expansion, organ growth, and stress responses (Vigneault et al., 2007; Agueci et al., 2012; Takatani et al., 2017). As for their role in rearrangement of microtubules during mitosis, NEK6 is known to associate with spindle and phragmoplast (Motose et al., 2011), but its function remains obscure.

## Protein Phosphatases Regulating Map Activity

The reversibility of phosphorylation is ensured by cooperation between kinases and phosphatases. Numerous protein phosphatases were found in plants, with Ser/Thr specific phosphoprotein phosphatases (PPPs) being a prominent group among them. PPPs encompass large number of proteins, which can be grouped in several protein families. Three of these families were implicated to regulate microtubule dynamics during cytokinesis (Samofalova et al., 2017).

Type one protein phosphatases (TOPPs, also called PP1s) were predicted to be part of cell cycle regulation (Farkas et al., 2007; Boyer and Simon, 2015), which is supported by finding putative CDK recognition sites (Kwon et al., 1997) as well as noting crucial role of animal PP1s in cell cycle progression (Rodrigues et al., 2015). However, the functions of these proteins were not comprehensively studied to this date.

More progress was achieved in solving the function of protein phosphatase type 2A (PP2A). These PPs consist of three subunits—scaffolding (A), regulatory (B), and catalytic (C). They were characterized in both monocots (Wright et al., 2009) and dicots (Camilleri et al., 2002). Moreover, their animal homologs were found to be essential for regulating microtubule structures in both meiosis and mitosis (Tang et al., 2016; Enos et al., 2017; Varadkar et al., 2017). In plants, PP2A controls organization and dynamics of both cortical and mitotic microtubules (Figure 1; Camilleri et al., 2002; Yoon et al., 2018). This view is supported by observing knockout mutants displaying abnormal arrangement of cortical microtubules and severe problems with PPB formation and cell division plane orientation (Torres-Ruiz and Jurgens, 1994; Traas et al., 1995; McClinton and Sung, 1997). During mitosis, PP2A forms a complex with tonneau 1 (TON1) and TON1 recruiting motif proteins (TRMs) (Spinner et al., 2013). TON1 and TRMs recruit this complex to site where PPB forms (Drevensek et al., 2012), and there the complex governs PPB development. As it remains at this site even after PPB disassembly, it is possibly involved in CPFS maintenance (Wright et al., 2009). Targets of PP2A-driven dephosphorylation could be MAPs marking cytokinetic structures (specifically MOR1, TON1, and CLASP; Twell et al., 2002; Kawamura, 2005; Xu et al., 2008; Ambrose et al., 2011). PP2A could temporally and spatially restrict common MAP activities and this would allow microtubule stabilization and formation of PPB (Walker et al., 2007; Wright and Smith, 2007; Lipka et al., 2015).

Metallo-dependent protein phosphatases (PP2C) might be part of cell division machinery as well, since knockout mutants display improper cell division orientation (Song et al., 2006). These phosphatases are also implicated in regulating MAPKs and CDK (Meskiene et al., 1998; Umbrasaite et al., 2010). Currently, their role in cortical microtubule rearrangement in response to environmental stimuli has been explored (Bhaskara et al., 2017; Qu et al., 2018). However, details on how PP2C is integrated into the regulatory network of cytokinesis remain undisclosed.

Although plenty of research has been done in elucidating the role of kinases in microtubule reorganization during cell cycle, phosphatases involved in these events remain largely understudied. The cause of this lies mainly in the fact that these phosphatases form multiprotein complexes, which is a serious challenge for both analysis and evaluation. Nevertheless, the current advances in understanding the role of PP2A in regulating microtubule dynamics shows it is not an impossible task.

## Conclusions/Future Directions

In summary, reversible phosphorylation of several different MAPs is essential for regulating microtubule dynamics and organization during the plant cell division. The affinity of MAPs for microtubules can be downregulated or restored pending on their phosphorylation status. To this extend, several protein kinases and phosphatases have been shown to target cytoskeletal proteins with various roles in the regulation of mitotic spindle and phragmoplast assembly and progression. However, many questions remain unanswered and are expected to be addressed in the near future:

1. How phosphorylation may affect microtubule nucleation during acentrosomal mitotic spindle formation?
2. Is phosphorylation related to the transition from PPB to mitotic spindle?
3. How phosphorylation really controls microtubule bundling during phragmoplast expansion?
4. Which phosphatases are reinstating microtubule binding of MAP65 proteins?
5. Which mechanisms allow the regulation of the same cytoskeletal proteins (e.g., MAP65-1, -2, and -3) by different protein kinases (e.g., MPK4 and MPK6 or auroras) with a different biological outcome (i.e., progression of cytokinesis and CDP orientation, respectively)?
6. How global phosphoproteomics analyses will help to decipher reversibly phosphorylatable cytoskeletal substrates in model cell suspension systems that can be synchronized?
7. How differential (phospho)proteomics comparing wild types with protein kinase/phosphatase mutants will advance our knowledge in the identification of cytoskeletal substrates?

## Author Contributions

TV drafted the manuscript and drawn Figure 1. JŠ contributed critical evaluation and editing of the text. GK conceived the topic, supervised TV during drafting of the manuscript and edited its final form together with JŠ.

### Conflict of Interest Statement

The authors declare that the research was conducted in the absence of any commercial or financial relationships that could be construed as a potential conflict of interest.
